# Diuretic Effect and Metabolomics Analysis of Crude and Salt-Processed Plantaginis Semen

**DOI:** 10.3389/fphar.2020.563157

**Published:** 2020-11-24

**Authors:** Chao Li, Rou Wen, De Wen Liu, Qiang Liu, Li Ping Yan, Jian Xiong Wu, Yi Jing Guo, Su Yun Li, Qian Feng Gong, Huan Yu

**Affiliations:** ^1^ School of Pharmacy, Jiangxi University of Traditional Chinese Medicine, Nanchang, China; ^2^ Institute of Chinese Materia Medica, China Academy of Chinese Medical Sciences, Beijing, China; ^3^ Department of Chemistry, Stanford University, CA, United States

**Keywords:** Plantaginis Semen (Cheqianzi), diuretic activity, metabolomics, saline processing, TCMS

## Abstract

Plantaginis Semen (PS) is well recognized in traditional Chinese medicine (TCM) and health products. Crude PS (CPS) and salt-processed CPS (SPS) are the two most commonly used decoction pieces of PS, and are included in the 2020 edition of *Chinese Pharmacopoeia*. Although they all have multiple effects, the mechanisms for treating diseases are different and remain unclear, the processing mechanism of SPS is also indeterminate, which hinders their clinical application to a certain extent. In order to solve these problems and further develop PS in the clinical application. Here, we used saline-loaded model rats for experiments, and utilized an integrated approach consisting of pharmacological methods and metabolomics, which could assess the diuretic impact of CPS and SPS ethanol extracts on saline-loaded rats and elucidate the underlying mechanism. The results showed that CPS and SPS both produced increased urine volume excretion and urine electrolyte excretion, but the levels of aldosterone (ALD) and aquaporin 2 (AQP2) were decreased. And 30 differential metabolites such as linoleic acid, lysoPC(O-18:0), sphingosine-1-phosphate, lysoPC(18:0) were found, mainly involving three metabolic pathways. In conclusion, CPS and SPS both have a diuretic effect, and that of SPS is better. This work investigated the possible diuretic mechanisms of CPS and SPS which may also be the mechanism of PS for anti-hypertension. In addition, a holistic approach provided novel and helpful insights into the underlying processing mechanisms of TCM.

## Introduction

Diuretics are potent therapeutic compounds that have been extensively employed for the treatment of chronic conditions such as hypertension and common edematous disorders including cardiac failure, hepatic cirrhosis, and nephrotic syndrome. The most frequently used diuretics include thiazides (e.g., hydrochlorothiazide), aldosterone (ALD) antagonists (e.g., spironolactone), and loop diuretics (e.g., furosemide). Furthermore, certain diuretics have also been historically used for the treatment of more specific diseases such as diabetes insipidus, glaucoma, hypercalcemia, hypercalciuria, and cerebral edema (Brater et al., 2000; Wilcox et al., 2002; Sarafidis et al., 2010; Ellison et al., 2019). Although diuretics provide important therapeutic effects in the treatment of various diseases similar to all potent therapeutic agents, its clinical use has also been associated with several adverse effects, such as increases in the risk of lip cancer (Pottegård et al., 2017), fracture risk in children with congenital heart disease (Heo et al., 2018) and acute risk of hip fracture in elderly individuals (Lai et al., 2017). Additionally, an increasing number of researches or studies have described the diuretic effects of traditional medicines. Furthermore, studies have focused on the positive effects of herbs and botanicals on health and wellness, in addition to their potential used in the treatment of some diseases (Wright et al., 2007).

*Plantago asiatica* L. (PAL) has historically been employed in Asian countries as traditional medicine as well as a dietary health supplement (Samuelsen, 2000; Kho et al., 2017; Li et al., 2017; Yoon et al., 2019). PAL is a TCM from plants of the Plantaginaceae family, which are widely distributed in Asian and European countries such as China, Korea, and Japan (Nishibe, 2002; Park et al., 2007). The dried mature seeds of PAL or *P. depressa* Willd (PDW) have been largely employed in TCM and are commonly called Plantaginis Semen (PS, Che Qian Zi). In addition, PAL, PDW, and PS are both Chinese medicines and health products listed by the National Health Commission of the People’s Republic of China (http://www.nhc.gov.cn/). Reference books in TCM describe PS as a diuretic agent that is employed to “promote water metabolism as well as purge dampness” (Gu, 2007). Cold-natured with a sweet flavor, PS exhibits effects that are localized in the kidney, small intestine, liver and lung meridians and functions to clear heat, promote diuresis, clear dampness, relieve thirst, enhance eyesight and remove phlegm, as described in the 2020 edition of *Chinese Pharmacopoeia*. Current pharmacological studies have revealed that PS possesses diuretic, anti-inflammatory, anti-oxidative, blood lipid-lowering, blood pressure-lowering, and immune regulatory actions (Pal et al., 2014). Medicinal plants of the *Plantago* genus, as are widely distributed, easily planted and low in price, having broad prospects in the clinical application (Feinglos et al., 2013). The compounds occurring in these species consist of phenylethanoid glycosides, guanidine derivatives, flavonoids, terpenoids, fatty acids, organic acids, etc. (Wang et al., 2016).

Crude PS (CPS) and salt-processed PS (SPS) are the two most common commercially available forms of PS, and these are described in the 2020 edition of *Chinese Pharmacopoeia*. Phytochemical investigations of PS have implied that terpenoids and phenylethanoid glycosides, particularly geniposidic acid, isoacteoside, and acteoside, are the most active components in CPS and SPS (Gu et al., 2016). According to TCM theories, the kidney plays the role of hosting and regulating the body’s water metabolism, and salt processing can increase the inclination and alter the action of PS, enhancing its effects in the kidney meridian and thereby strengthening its efficacy in diuresis (Gong, 2012). Furthermore, we have determined that 148 prescriptions in the Yaozhi Database (https://www.yaozh.com/, is one of the mainstream medical databases in China) contained PS, whereas 24 prescriptions utilized SPS in promoting diuresis, treating strangury and clearing heat, such as Wuzi Yanzong Wan and Jinkui Shenqi Wan. Some of them also included CPS, such as Jinsha Wulin Wan and Jisheng Shenqi Wan, which commonly are used in the treatment of difficult urination, edema, and dampness-heat. In clinical practice, CPS has traditionally been employed by various countries as diuretic, antipyretic, and expectorant (Wang et al., 2016). However, only one report on the diuretic effect of PS was found in PubMed, and that study did not assess its diuretic effect on urinary output or sodium excretion (Doan et al., 1992). Whether CPS has a diuretic effect remains in doubt. On the other hand, SPS also shows potential value in tonifying the kidney, which may be due to salt processing (Zou et al., 2015). Only a few pharmacological mechanism-related studies on PS and SPS concerning diuretic effects have been conducted to date.

TCMs and their prescriptions exhibit advantages in the treatment and prevention of diseases from multicomponent, multichannel, and multitarget (Wang et al., 2012). However, the lack of information on the chemical composition of TCM materials, including their bioactive components, mechanism of action, therapeutic targets, and drug metabolism, has hindered progress in the use of TCMs (Wang et al., 2019). The systemic approach of metabolomics has helped to elucidate the mechanism of action and the effects of TCMs (Lin et al., 2019).

In this study, under the direction of literature references and preliminary experiments of PS and SPS, the role and mechanism of CPS and SPS for the treatment of saline-loaded rats was investigated by an untargeted metabolomics approach. To begin with, urinary excretion and electrolyte excretion were measured to explore the diuretic effects of CPS and SPS. Then, with the help of histopathological examination, the effect of CPS and SPS on rat kidneys was observed. Third, the expression of aquaporin 2 (AQP2), and ALD were measured to discover whether CPS and SPS had any effect on these substances. Moreover, metabolomics analysis using ultra-performance liquid chromatography-quadrupole time-of-flight mass spectrometry (UPLC-Q-TOF/MS) was performed to identify and relatively measure serum metabolite levels. In addition, multivariate data analysis and pathway analysis were used to investigate the underlying diuretic and processing mechanisms of CPS and SPS.

## Materials and Methods

### Reagents and Animals

PS (commonly known as Che Qian Zi) specimens were gathered from Jiangxi Guhan Refined Chinese Herbal Pieces Co., Ltd. (Jiangxi, China) and were identified as the mature seed of *P. asiatica* L. by Professor Q. F. Gong of Jiangxi University of Traditional Chinese Medicine in Nanchang, China. Relevant testing indicators were following the 2020 edition of *Chinese Pharmacopoeia*. A voucher specimen (NO20180701) was deposited at the School of Pharmacy, Jiangxi University of Traditional Chinese Medicine, Nanchang, China. Formic acid (purity: 99%, LC-MS grade) was obtained from Thermo Fisher Scientific Corp. (MA, United States), methanol (LC-MS grade) and acetonitrile (LC-MS grade) were bought from Merck KGaA (Darmstadt, Germany), and purified water from a Milli-Q-Plus system (Millipore, Bedford, MA, United States) was used. Furosemide (Jiangsu Hailin Pharmaceutical Corporation, Jiangsu, China). Chemical reference substances were purchased from Chengdu Chroma-Biotechnology Co., Ltd. (Chengdu, China), and the catalog numbers are CHB171229 (geniposidic acid, ≥98%), CHB171103 (verbascoside, also named acteoside, ≥98%), CHB170612 (isoverbascoside, also named isoacteoside, ≥98%). Enzyme-linked immunosorbent assay (ELISA) kits of ALD and AQP2 were obtained from Hua Mei Biological Engineering Co., Ltd. (Wuhan, China), and the catalog numbers are CEA911Ge and CSB-E08243r. All other reagents and chemicals were of analytical grade.

A total of 40 male Sprague-Dawley rats (weighing 160–180 g) were purchased from Hunan SJA Laboratory Animal Co., Ltd. (Changsha, China) (License NO. SYXK 2017-0004). Rats were maintained in polyethylene boxes with a controlled room temperature (22 ± 2)°C and a 12 h light-dark cycle and provided autoclaved water and food *ad libitum*. All of the experiments were performed following the guidelines established by the Institutional Animal Care and Use Committee of China and duly approved by the Animal Care and Research Committee of Jiangxi University of Traditional Chinese Medicine (Nanchang, China) under license No. JZLLSC2020_0108.

### Preparation of Crude Plantaginis Semen and Salt-Processed Crude Plantaginis Semen

SPS was prepared according to the 2015 edition of *Chinese Pharmacopoeia*. The specific processing method was as follows: CPS was put into a frying container, lightly heated and fried until it made a slight popping sound and released an aroma, then the salt sprayed evenly on the surface of the medicinal material, continued to fry it until dried, after which it was allowed to cool.

CPS and SPS were separately pulverized and then filtered with No. 3 sieve of pharmacopoeia standard, then the decoction pieces (250 g, respectively) of CPS and SPS were extracted three times with four times volume of 80% ethanol under reflux extraction. After filtering the extract, the solvent was evaporated to dryness and the residue was resuspended in saline solution (0.9% NaCl, w/v). The solutions of 0.5 g·mL^−1^ CPS and 0.5 g·mL^−1^ SPS were obtained, and a furosemide solution (a positive control drug) with a concentration of 1 g·L^−1^ was also prepared with a physiological saline solution.

### Content Determination of Main Components in Crude Plantaginis Semen and Salt-Processed Crude Plantaginis Semen

UPLC was performed with an ACQUITY UPLC H-Class Ultra-Performance Liquid Chromatograph [Waters, United States, including diode array (PDA) detector and Empower 3 chromatography workstation], and separation was achieved on a Waters ACQUITY UPLC C_18_ column (2.1 mm × 100 mm, 1.7 μm), the chromatographic conditions included mobile phases consisting of methanol (A) and 0.1% formic acid solution (B). The gradient program was as follows: 0–1 min, 5%–10% A; 1–2 min, 10%–20% A; 2–10 min, 20%–35% A; 10–18 min, 35%–50% A; 18–19 min, 50% A; and 19–20 min, 5% A. The column temperature was 30°C, the flow rate was 0.4 ml·min^−1^, the injection volume was 2 μL, and the detection wavelength was 254 nm. Quantification was performed by generating calibration curves for every compound based on standards.

### Diuretic Activity

The diuretic activity was determined following previously reported methods (Zhao et al., 2009; Zhao et al., 2012; Feng et al., 2013; Chen et al., 2014). Rats were fasted but allowed access to water for 12 h before testing. Their urinary bladders were emptied by gentle compression of the pelvic area and by pulling of their tails. Each of the rats was then orally administered 5 ml·100 g^−1^ body weight (BW) of isotonic saline (0.9% NaCl, w/v) to introduce a uniform water load. Thirty minutes later, these rats were randomly assigned to five groups (*n* = 8 per group) and treated orally in the following manner: model (M) group, 2 ml·100 g^−1^ BW of isotonic saline; normal control (NC) group, no medication and saline, other measures consisted with remaining groups; CPS group, 2 ml·100 g^−1^ BW of CPS; SPS group, 2 ml·100 g^−1^ BW of SPS; positive group, 2 ml·100 g^−1^ BW of furosemide. The rats were individually housed in metabolic cages, and cumulative urine output for 6 h was determined. The concentrations of electrolytes (i.e., Na^+^, K^+^, Cl^−^) of the 6-h rate urine samples were determined.

### Sample Collection and Preparation

Treatments were administered orally and continuously for 8 days. On the last day, 10% chloral hydrate (0.3 ml·100 g^−1^) was intraperitoneally injected to anesthetize the rats. Then, blood samples were collected by venipuncture of the aorta abdominalis and into 10 ml Eppendorf (EP) tubes, whereas kidney tissues were isolated. The blood samples were allowed to stand for 30 min and then centrifuged at 4,000 r·min^−1^ for 10 min at 4°C to isolate the sera, which were then separated into three equal parts and stored at −80°C in order to further use. Subsequently, the right kidney of each rat was resected and fixed in 10% paraformaldehyde for histological analysis. The left kidney of each rate was immediately washed with hypothermic normal saline solution after isolation and preserved at −80°C until ELISA analysis.

Prior to extraction, the frozen samples were thawed out at 4°C. Approximately 100 µL of each plasma sample was mixed with methanol (600 µL), vortexed for 2 min, next the protein precipitate was isolated after centrifuged at 4,000 r·min^−1^ for 15 min. Before mixed with 100 µL of methanol that the supernatant was evaporated to dryness under nitrogen. Centrifuged at 14,000 r·min^−1^ for 10 min at 4°C and filtered through a 0.22 μm microporous membrane filter. Then, the supernatant was subjected to UPLC-Q-TOF/MS analysis.

Quality control (QC) samples were prepared by mixing equal volumes of every sample. Then, we performed three consecutive injections of the QC samples before testing. One blank mobile phase sample and one QC sample were tested after every eight samples.

### Ultra-Performance Liquid Chromatography-Quadrupole Time-of-Flight Mass Spectrometry Analysis

The serum samples were analyzed on a Triple TOF™ 5600 (AB Sciex, Foster City, CA, United States) LC-MS instrument with a DuoSpray™ ionization source (AB Sciex, Foster City, CA, United States). Chromatographic separation was achieved by using a Prominence™ UPLC system (Shimadzu, Japan) that included a CBM-20A automatic controller, a SIL-30 AC autosampler, a CTO-30 AC column oven, a DGU-20A3 vacuum degasser, and an LC-30 CEAD infusion pump. The data quality was calibrated by DUO Spray™ ion source using CDS (Automated calibrant delivery system, AB Sciex, Foster City, CA, United States). The ionization source is connected to the AB sciex automatic calibration system (CDS), and it is automatically calibrated once every 10 samples, and the calibration solution flow rate is 0.35 ml/min.

The prepared serum samples were sequentially injected into an ACE EXCEL C_18_ column (2.1 mm × 100 mm, 2 µm) with a column temperature of 40°C, and flow rate was retained at 0.3 ml·min^−1^. The mobile phase consisted of 0.1% formic acid solution (A) and acetonitrile (B). The optimized UPLC elution program was as follows: 0–8 min, 5–40% B; 8–30 min, 40–95% B; 30–32 min, 95% B.

The mass spectrometer was equipped with an electrospray ionization (ESI) operating in positive and negative ion modes. The scanning range of the TOF-MS was *m*/*z* 100–1,500. The mass spectrometer parameters were set as follows: ion spray voltage, 5,500/4,500 V; drying gas, N_2_; gas temperature, 500°C; nebulizer gas 1, 50 psi; heater gas 2, 50 psi; curtain gas, 40 psi; declustering potential, 100/−100 V; collision energy, 35/−35 eV; and collision energy spread, 15 eV.

### Quantitative Estimation of Serum Aquaporin 2 and Aldosterone

AQP2 is a membrane-associated water channel within the kidney collecting duct that regulates urine volume (Cheung et al., 2020; Susanna, 2020). Thus, the AQP2 content was determined in the kidney grinding fluid of each group of rats. ALD is the major hormone that maintains water content in the body via the antidiuretic activity of the kidney (Gonzalez et al., 2020). The determination of the ALD content in rat serum was conducted. Therefore, related ELISA kits were selected for further verification. The kits were prepared for AQP2 and ALD detection (Huamei Company, Wuhan, China) following the manufacturer’s protocols, and measurements were conducted spectrophotometrically at 460 nm. According to the following standard curves, the sample concentration was calculated: *Y*_AQP2_ = 120.34*X* ^2^ + 263.07*X* − 0.929 5 (*R* ^2^ = 0.9997) and *Y*_ALD_ = −230.032 3 + 258.601 7*X* ^−1^ (*R* ^2^ = 0.9991).

### Biomarker Screening and Identification

Raw data gathered by the LC-MS system were input into MarkerView™ software to preprocess the data. Chromatographic peak alignment and normalization were initially performed. Mass tolerance was 10 ppm, retention time ranged from 1 to 34 min, quality scan ranged from *m*/*z* 100–1,000, whereas other parameters were set to default. The number of extracted ions was standardized using the total peak area method, which was normalized using the sum of the total area. Significantly different variables (*p* < 0.05) were selected. The screened ion information was saved in an EXCEL text format, imported to SIMCA version 14.1 for principal component analysis (PCA), then analyzed using orthogonal partial least squares discriminant analysis (OPLS-DA). Generally selected the metabolites in the range of |*p*(corr)| > 0.5, |*p*| > 0.1, the selected variables showed high reliability and magnitude in the S-plot based on the OPLS-DA models. Features exhibiting *t*-test *p* < 0.05 were cross-referenced to public databases such as the Human Metabolome Database (HMDB), Small Molecular Pathway Database (SMPD), and METLIN. The 2D molecular structures of the identified metabolites were downloaded, and subsequently, the relevant UPLC-Q-TOF/MS data information was imported into PeakView version 1.2. The corresponding *m*/*z* and retention time were compared, and the secondary mass spectra were compared with the chemical structures. The selected biomarkers were identified by a comparative analysis of the results of qualitative analysis and secondary mass spectra, followed by confirmation of the results using PeakView version 1.2 (*δ* < 10 ppm). Finally, candidate compound pathway analysis was performed used the available online database MetaboAnalyst 4.0 (https://www.metaboanalyst.ca/).

### Statistical Analysis

The results are expressed as the mean ± standard deviation. Multiple comparisons were performed using ANOVA, followed by student’s *t*-test. Differences with *p* < 0.05 or <0.01 between the M and drug treatment groups were deemed statistically significant.

## Results

### Ultra-Performance Liquid Chromatography Chromatograms of Crude Plantaginis Semen and Salt-Processed Crude Plantaginis Semen Extracts

The main compounds geniposidic acid, verbascoside, and in CPS and SPS were analyzed by using UPLC chromatographic. Supplementary Figure S1 showed the results of the chromatogram. Using plots consisting of peak area (*Y*) vs. concentration (*X*, mg·L^−1^), the regression equations of the three major components as well as their correlation coefficients (*R* ^2^) were determined follows: geniposidic acid, *Y* = 1.08 × 10^6^ *X* − 2,660 (*R* ^2^ = 0.999 9); verbascoside, *Y* = 2.21 × 10^6^ *X* − 2,290 (*R* ^2^ = 0.999 9); isoverbascoside, *Y* = 2.41 × 10^5^ *X* − 1,250 (*R* ^2^ = 0.999 9). The contents of the three main compounds in CPS and SPS were seen in Table 1. The content of the isoverbascoside of SPS is about three times that of CPS, and geniposidic acid and verbascoside also showed a certain increase. The result of UPLC chromatogram was shown in Supplementary Figure S1.

**TABLE 1:** Results of content determination of samples.

Format	Geniposidic acid (mg·g^−1^)	Verbascoside (mg·g^−1^)	Isoverbascoside (mg·g^−1^)
CPS	9.41	4.67	0.89
SPS	11.25	5.14	2.65

**TABLE 2:** Effect of 8-day oral doses of CPS and SPS sclerotia extracts and furosemide on 6 h urinary electrolyte excretion of Na^+^, K^+^, and Cl^−^ in saline-loaded rats.

Group^a^	Dose (g·kg^−1^ BW)	Urinary electrolyte concentration (mmol/L)		
		Na^+^	K^+^	Cl^−^
M	—	88.70 ± 5.00	81.51 ± 9.74	124.57 ± 17.45
NC	—	—	—	—
CPS	10	111.24 ± 3.68^**^	105.51 ± 11.80^**^	176.52 ± 12.01^**^
SPS	10	118.23 ± 8.12^**#^	106.52 ± 12.76^**^	180.28 ± 19.03^**^
Furosemide	0.01	124.23 ± 6.59^**##^	96.03 ± 12.61^*^	182.22 ± 23.24^**^

Values are presented as the mean ± SD of eight rats in every group. *p < 0.05; **p < 0.01 relative to the M group using LSD and ANOVA; ^#^ *p* < 0.05; ^##^ *p* < 0.01 relative to CPS using LSD and ANOVA.

a All rats were orally pretreated with 5 ml·100 g^−1^ BW normal saline before test substance administration.

### Evaluation of Diuretic Effects

**FIGURE 1:**
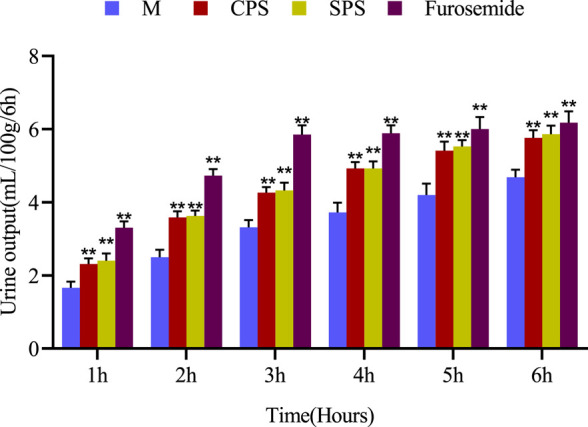
The effect of single oral administration of CPS, SPS and furosemide on the urine output of saline-loaded rats. Urine output was measured at 1, 2, 3, 4, 5, and 6 h post-administration. The cumulative values are expressed as the mean ± SE of eight rats in every group. *p < 0.05; **p < 0.01 compared to the M group using the Student’s *t*-test.

The results obtained after the oral administration of the corresponding medicine to rats are shown in Figure 1. Compared with the M group, rats given furosemide depicted a significant increase in urine volume output within 6 h (*p* < 0.01) (Figure 1); the administration of CPS and SPS ethanol extractions significantly increased urine output within 6 h relative to the M group (*p* < 0.01) and had a diuretic effect. The average urine output was higher for SPS than for CPS after administration of at the same concentration. From the perspective of urine electrolytes, compared to the M group, the furosemide group showed a significant increase in urine Na^+^, K^+^, and Cl^−^ excretion (*p* < 0.05, *p* < 0.01). The urine electrolyte excretion of rates administered ethanol extracts of CPS and SPS also increased significantly within 0–6 h (*p* < 0.01). Comparing the administration of ethanol extracts at the same concentration, it could be seen that CPS was slightly weaker than SPS based on urine See Table 2.

### Aquaporin 2 and Aldosterone Concentrations

**FIGURE 2:**
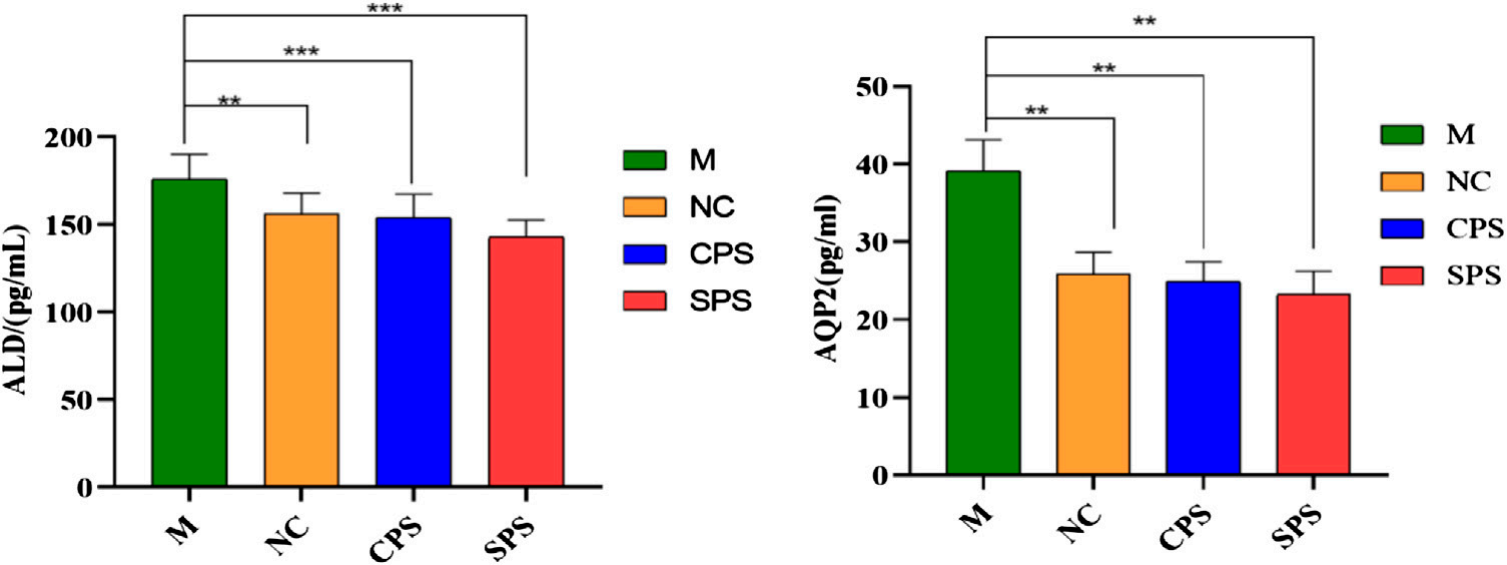
The effects of different treatment methods on the ALD of serum and AQP2 of kidney in saline-loaded rats (pg·L^−1^, mean ± SD, *n* = 8). Note: compared to the M group, **p < 0.05 and ***p < 0.001.

The relative ALD and AQP2 contents after 8 days of CPS and SPS extract treatment in normal rat serum were found to be downregulated compared to the model group (*p* < 0.01, *p* < 0.001) (Figure 2). Relevant factor indices of the kidney and serum are presented in Figure 1. Excess saline in the stomach still affected rats. The levels of ALD and AQP2 in the CPS and SPS groups markedly decreased after drug treatment relative to the saline-loaded model group (*p* < 0.01). Although there was no significant difference between the relative contents of ALD and AQP2 in the CPS and SPS groups, as determined with the mean ± SD, the effect of SPS was slightly stronger than that of CPS. The results showed that both CPS and SPS improved saline loading, and SPS possibly had better effects than CPS.

### Effects of Crude Plantaginis Semen and Salt-Processed Crude Plantaginis Semen on the Histopathological Morphology of Rat Kidneys

**FIGURE 3:**
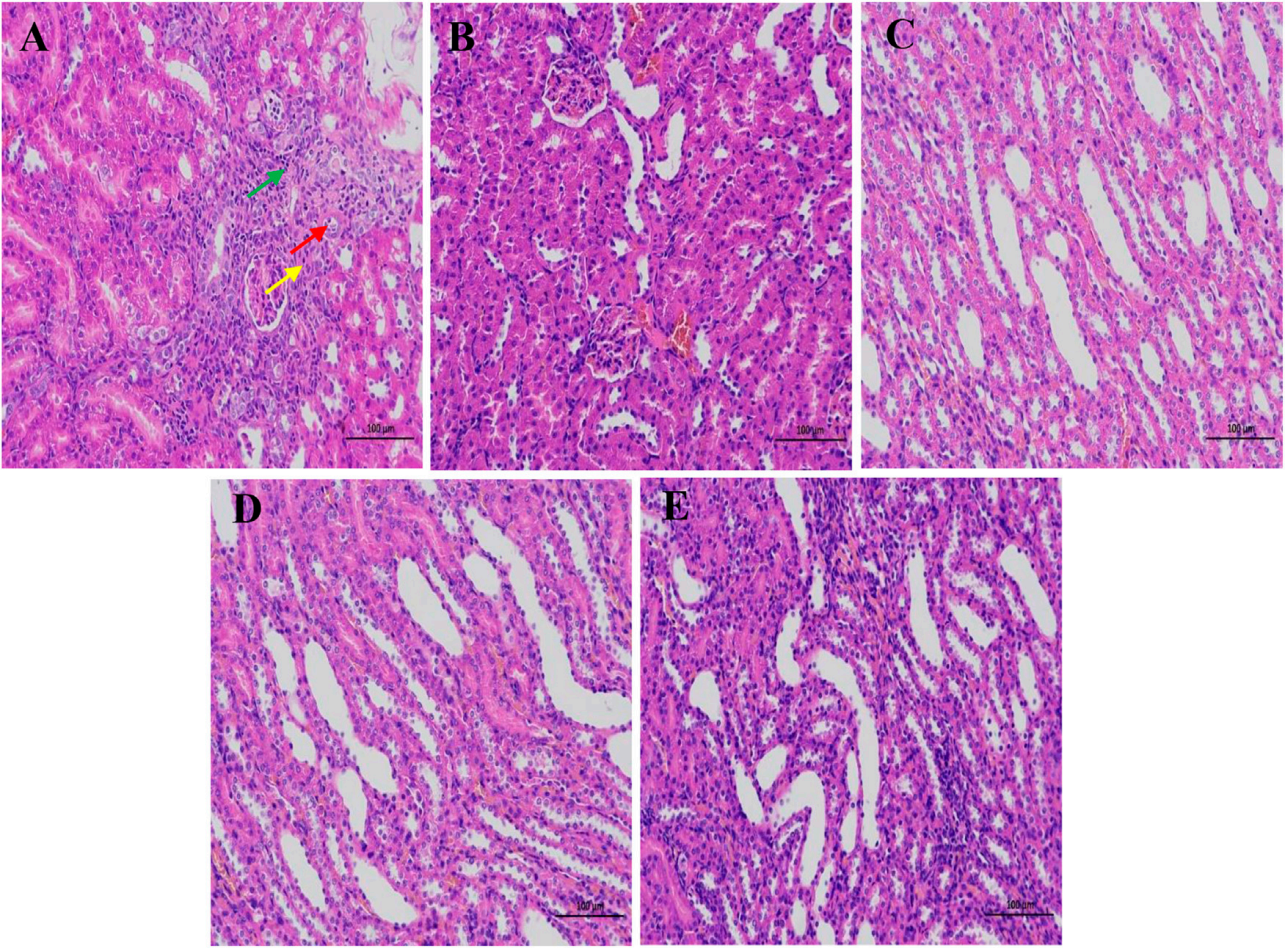
The pathological changes of the kidney tissue of rats in different treatments. **(A–E)** represent the M group, NC group, CPS group, SPS group, and furosemide group, respectively. Red arrows show local cortical renal tubular atrophy, lumen stenosis, and epithelial cell volume reduction. Yellow arrows show interstitial connective tissue hyperplasia, and green arrows show a small amount of lymphocyte infiltration. Scale bar = 100 μm.

Kidney tissues were stained with hematoxylin-eosin (HE) after fixation in 4% paraformaldehyde for 24 h. Figure 3 showed the absence of significant damage to the kidney tissues of normal rats. In addition, skin tissue and medulla were demarcated, renal tubules were abundant and closely arranged, and some renal tubules and collecting ducts were dilated at the junction of the cortex and medulla. The kidney sections of the saline-loaded model rats showed signs of renal tubular atrophy, lumen stenosis, and a small amount of lymphocyte infiltration. Treatments with CPS, SPS, and furosemide expanded the number of renal tubules and collecting ducts and markedly reduced the severity of histopathological changes.

### Metabolomic Results

#### Principal Component Analysis of Rat Serums Metabolomics Data

**FIGURE 4:**
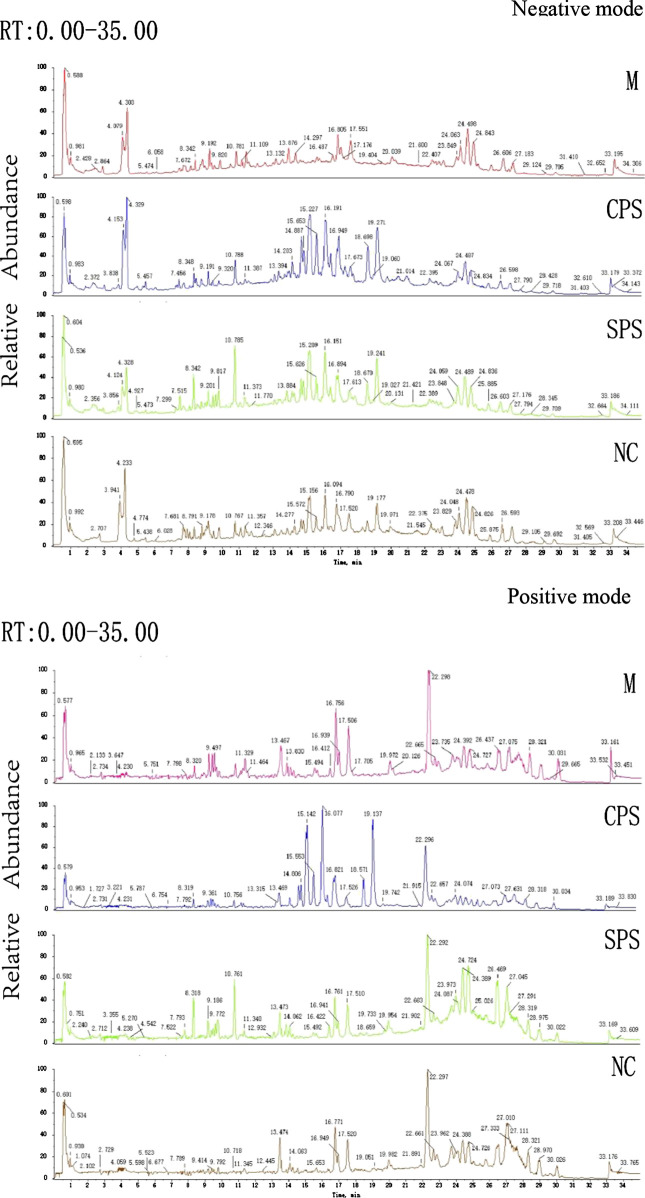
Total ion chromatograms of the M, CPS, SPS, and NC groups in negative model and positive mode.

**FIGURE 5:**
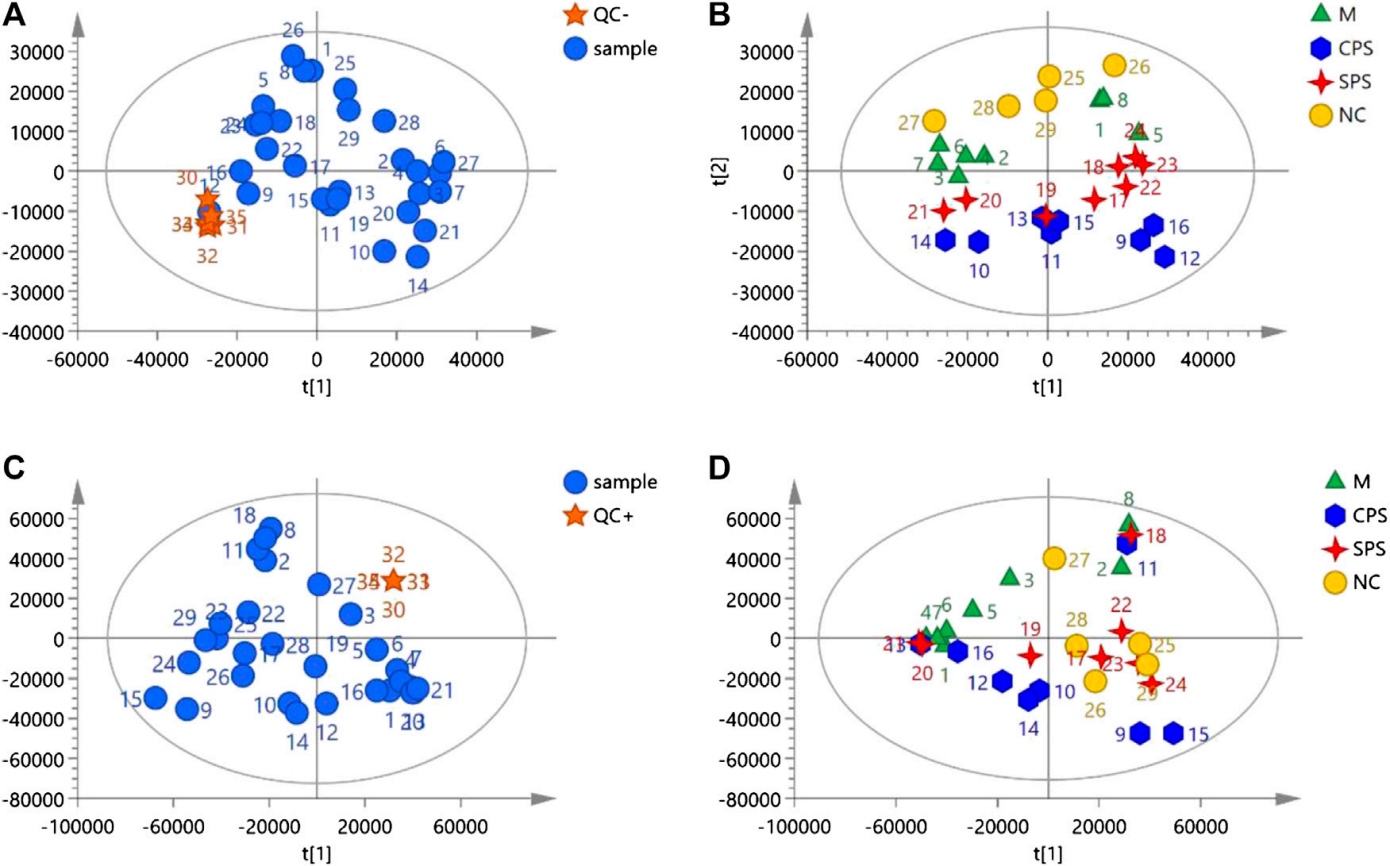
The negative and positive modes of quality control samples (QCs) evaluation and PCA plots of data acquired. **(A)** QCs in negative mode; **(B)** PCA plot of all samples analyzed in negative mode; **(C)** QCs in positive mode; and **(D)** PCA plot of all samples analyzed in positive mode.

Metabolomics data on the total ion current after C_18_ separation were obtained (Figure 4). The complexity of UPLC-Q-TOF/MS data is high due to a large number of variables, and PCA can obtain more information by reducing the number of variables via dimensionality reduction. In metabolomics research based on UPLC-Q-TOF/MS technology, to obtain reliable and high-quality data, analytical errors should not affect the results of multivariate statistical analysis. Multivariate analysis is used to monitor the stability and repeatability of actual samples and the projected aggregation of QC samples interspersed throughout the analysis batch on the principal component score map. A PCA score map showed that the stability of the established analytical method was good, and the differences between the test samples mainly came from the differences of the metabolites in the samples rather than from errors from the analytical method (QC) (Figures 5A, C). In negative ion mode, the model was autofitted using five components, which explained 54.5% of the variables in all groups. Although the clustering of each group was not obvious, the general trend of each group was shown in the PCA score plot. The NC group was notably separated from the model group, and the CPS and SPS groups clustered away from the model group (Figure 5B). In the positive mode, all groups were autofitted to the three components, which explained 65.3% of the variables. The PCA score plot revealed that the model group is distinct to the NC group, and the NC, CPS, and SPS groups tended to cluster at the bottom relative to the M group (Figure 5D).

#### Orthogonal Partial Least Squares Discriminant Analysis of Rat Serums Metabolomics Data

Different from PCA, OPLS-DA is a statistical method of supervised discriminant analysis. This method uses partial least squares regression to establish the relationship model between the metabolite expression level and the sample type to achieve the prediction of the sample type. To explore the alterations *in vivo* with the administration of CPS and SPS to saline-loaded rats and to elucidate the mechanism of action of PS (including CPS and SPS) from a metabolic perspective, this study introduced supervised OPLS-DA patterns. To prevent pattern overfitting, a typical seven-round cross-validation was utilized during pattern establishment, and permutation tests were employed for OPLS-DA pattern estimation. OPLS-DA was used to obtain a scatter plot of the predicted principal components (to [1]) and orthogonal principal components (to [1]) between each group. A distinct separation of the two groups within the OPLS-DA score plot was observed.

**FIGURE 6:**
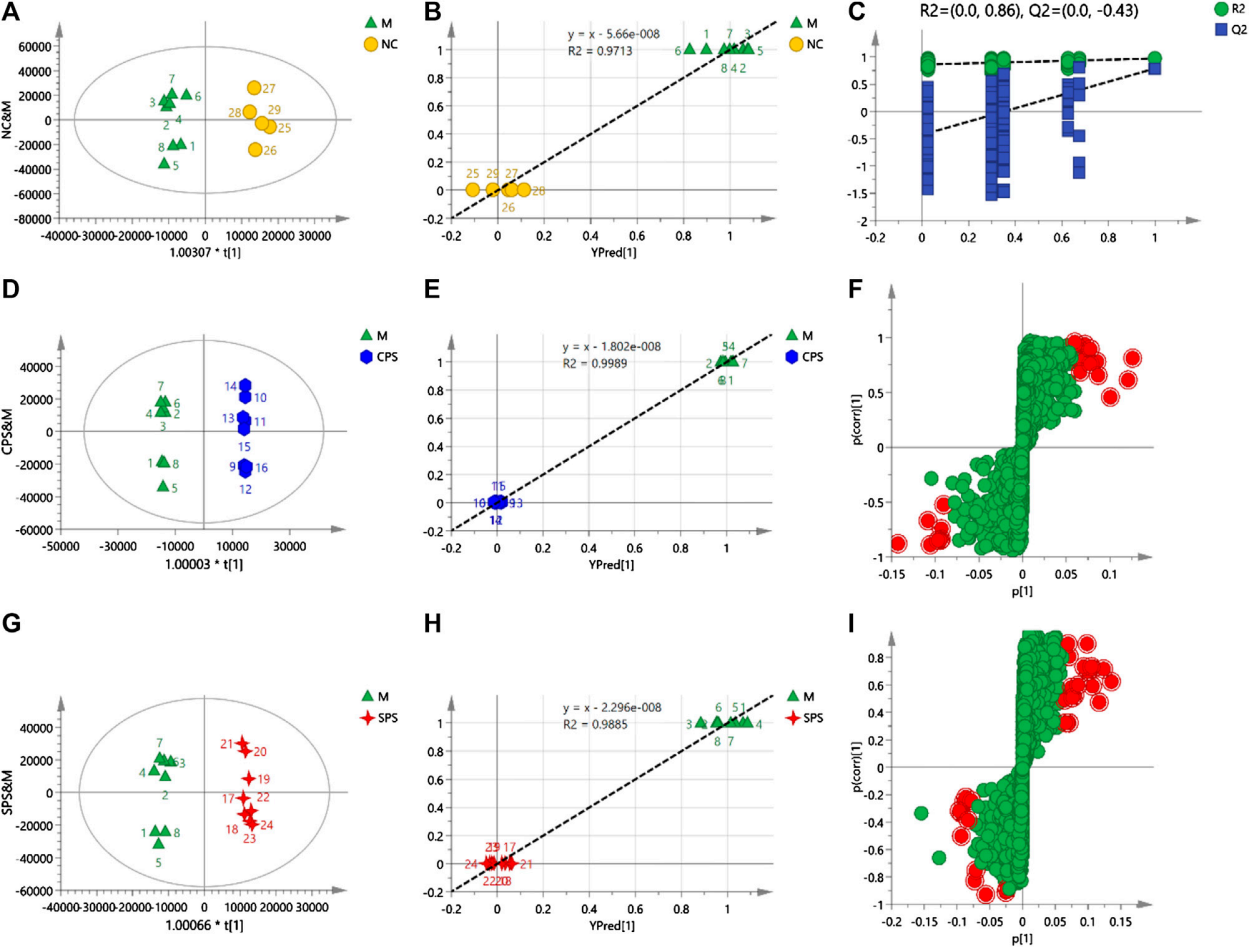
**(A)** Score plots of NC vs. M, **(B)** observation vs. predicted plots (NC and M), **(C)** permutation tests (NC and M), **(D)** score plots of CPS vs. M, **(E)** observation vs. predicted plots (CPS and M), **(F)** S-plots of CPS and M, **(G)** score plots of SPS vs. M, **(H)** observation vs. predicted plots (SPS and M), and **(I)** S-plots SPS and M. The acquisition of these figures based on using OPLS-DA in negative mode.

In the negative mode, a 1 + 2 + 0-component OPLS model was obtained between the NC and M groups. A distinct separation was observed between the model and NC groups when the parameters *R* ^2^ *X* and *Q* ^2^ > 0.5 (*R* ^2^ *X* = 0.732 and *Q* ^2^ = 0.789), indicating that the patterns were of superior explanatory and predictive ability. For the 1 + 4 + 0-component OPLS model, the CPS and model groups (*R* ^2^ *X* = 0.821 and *Q* ^2^ = 0.952) depicted two clusters for CPS and M group data, which indicated that this was a stable model. Notable discrepancies between the SPS and M groups were also observed, showing *R* ^2^ *X* = 0.759 and *Q* ^2^ = 0.825, and a 1 + 3 + 0-component OPLS model was obtained between the SPS and Figure 6.

**FIGURE 7:**
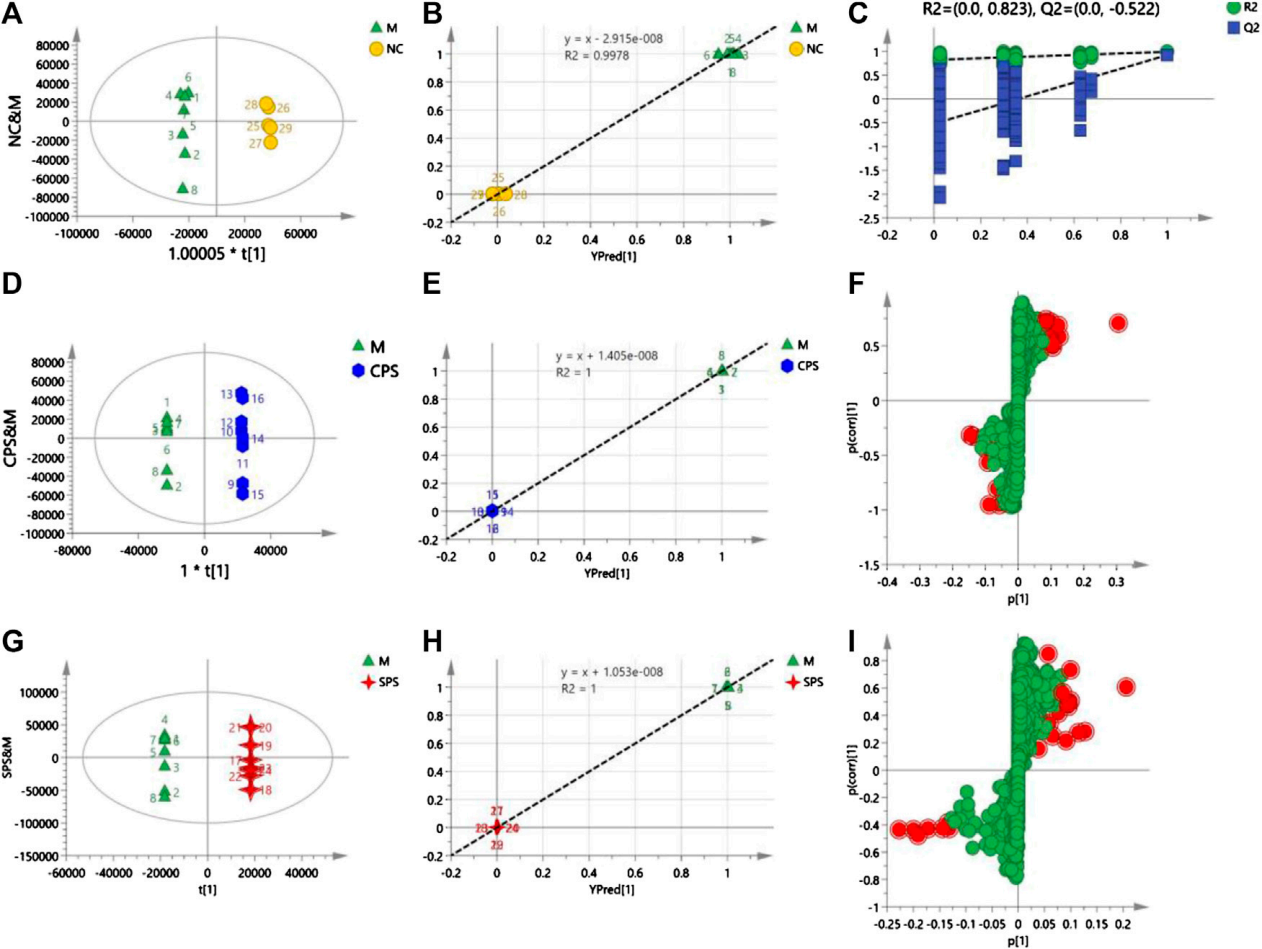
**(A)** Score plots of NC vs. M, **(B)** observation vs. predicted plots (NC and M), **(C)** permutation tests (NC and M), **(D)** score plots of CPS vs. M, **(E)** observation vs. predicted plots (CPS and M), **(F)** S-plots of CPS and M, **(G)** score plots of SPS vs. M, **(H)** observation vs. predicted plots (SPS and M), and **(I)** S-plots of SPS and M. The acquisition of these figures based on using OPLS-DA in positive mode.

In the positive mode, a 1 + 4-component OPLS model was observed between the M and NC groups, with *R* ^2^ *X* = 0.926 and *Q* ^2^ = 0.756, which verified the stability and reliability of the model. A 1 + 5-component OPLS model of M and CPS groups yielded values of *R* ^2^ *X* = 0.934 and *Q* ^2^ = 0.85. The plot of observed vs. predicted values revealed that the M and CPS groups were clustered separately and adjacent to the regression line. In addition, these results showed that the fitted OPLS model of the M and CPS groups is stable. The *R* ^2^ *X* and *Q* ^2^ values for the 1 + 6-component OPLS model of the model and SPS groups were 0.971 and 0.798, respectively, which showed that the model was Figure 7.

After verifying the stability and reliability of the model, variable importance in the projection (VIP) values were calculated by software for all metabolites (variables) in each OPLS-DA model. Variables were regarded as distinguishing a group when the VIP value >1 and the *p* < 0.05. For screening of metabolic markers (Table 3).

**TABLE 3:** Identification of serum metabolites.

No.	*m*/*z*	*t*_R_ (min)	Name	Formula	HMDB	Recall effect	Adduct
1	218.1033	2.24	Pantothenic acid	C_9_H_17_NO_5_	HMDB0000210	↓	[M − H]−
2	253.2171	23.83	Palmitoleic acid	C_16_H_30_O_2_	HMDB0003229	↓	[M − H]−
3	255.2346	26.58	Isopalmitic acid	C_16_H_32_O_2_	HMDB0031068	↓	[M − H]−
4	281.2484	27.19	Vaccenic acid	C_18_H_34_O_2_	HMDB0003231	↓	[M − H]−
5	277.2177	22.79	Linolenelaidic acid	C_18_H_30_O_2_	HMDB0030964	↓	[M − H]−
6	279.2344	24.85	Linoleic acid	C_18_H_32_O_2_	HMDB0000673	↓	[M − H]−
7	391.2841	12.51	3b,12a-Dihydroxy-5a-cholanoic acid	C_24_H_40_O_4_	HMDB0000348	↓	[M − H]−
8	407.2790	9.83	Allocholic acid	C_24_H_40_O_5_	HMDB0000505	↓	[M − H]−
9	436.2806	16.82	LysoPE(P-16:0/0:0)	C_21_H_44_NO_6_P	HMDB0011152	↑	[M − H]−
10	405.2613	8.70	3-Oxocholic acid	C_24_H_38_O_5_	HMDB0000502	↓	[M − H]−
11	407.2790	9.83	Muricholic acid	C_24_H_40_O_5_	HMDB0000865	↑	[M − H]−
12	457.2346	25.40	1-Lyso-2-arachidonoyl-phosphatidate	C_23_H_39_O_7_P	HMDB0012496	↑	[M − H]−
13	595.2871	21.65	Asparagoside B	C_33_H_56_O_9_	HMDB0029315	↓	[M − H]−
14	256.2621	22.92	Palmitic amide	C_16_H_33_NO	HMDB0012273	↓	[M + H]^+^
15	299.2938	27.42	Methyl stearate	C_19_H_38_O_2_	HMDB0034154	↓	[M + H]^+^
16	302.3069	11.18	Sphinganine	C_18_H_39_NO_2_	HMDB0000269	↓	[M + H]^+^
17	343.2627	28.30	DG(18:0/20:3(8Z,11Z,14Z)/0:0)	C_41_H_74_O_5_	HMDB0007169	↓	[M + H + K]^+^
18	380.2557	13.11	Sphingosine-1-phosphate	C_18_H_38_NO_5_P	HMDB0000277	↓	[M + H]^+^
19	480.3457	16.95	LysoPC(P-16:0)	C_24_H_50_NO_6_P	HMDB0010407	↓	[M + H]^+^
20	494.3229	14.11	LysoPC(16:1(9Z))	C_24_H_48_NO_7_P	HMDB0010383	↑	[M + H]^+^
21	496.3384	15.55	LysoPC(16:0)	C_24_H_50_NO_7_P	HMDB0010382	↓	[M + H]^+^
22	506.3614	15.65	LysoPC(P-18:1 (9Z))	C_26_H_52_NO_6_P	HMDB0010408	↓	[M + H]^+^
23	510.3903	19.97	LysoPC(O-18:0)	C_26_H_56_NO_6_P	HMDB0011149	↓	[M + H]^+^
24	508.3758	17.51	LysoPC(P-18:0)	C_26_H_54_NO_6_P	HMDB0013122	↓	[M + H]^+^
25	522.3553	16.86	LysoPC(18:1(11Z))	C_26_H_52_NO_7_P	HMDB0010385	↑	[M + H]^+^
26	524.3691	18.60	LysoPC(18:0)	C_26_H_54_NO_7_P	HMDB0010384	↑	[M + H]^+^
27	534.3908	18.38	LysoPC(20:0)	C_28_H_58_NO_7_P	HMDB0010390	↓	[M + H-H_2_O]^+^
28	538.3860	20.83	LysoPE(0:0/22:0)	C_27_H_56_NO_7_P	HMDB0011490	↑	[M + H]^+^
29	550.3846	19.75	PC(18:1(9Z)e/2:0)	C_28_H_56_NO_7_P	HMDB0011148	↓	[M + H]^+^
30	562.4255	21.47	LysoPC(22:0)	C_30_H_62_NO_7_P	HMDB0010398	↓	[M + H-H_2_O]^+^

#### Identification of Characteristic Metabolites

**FIGURE 8:**
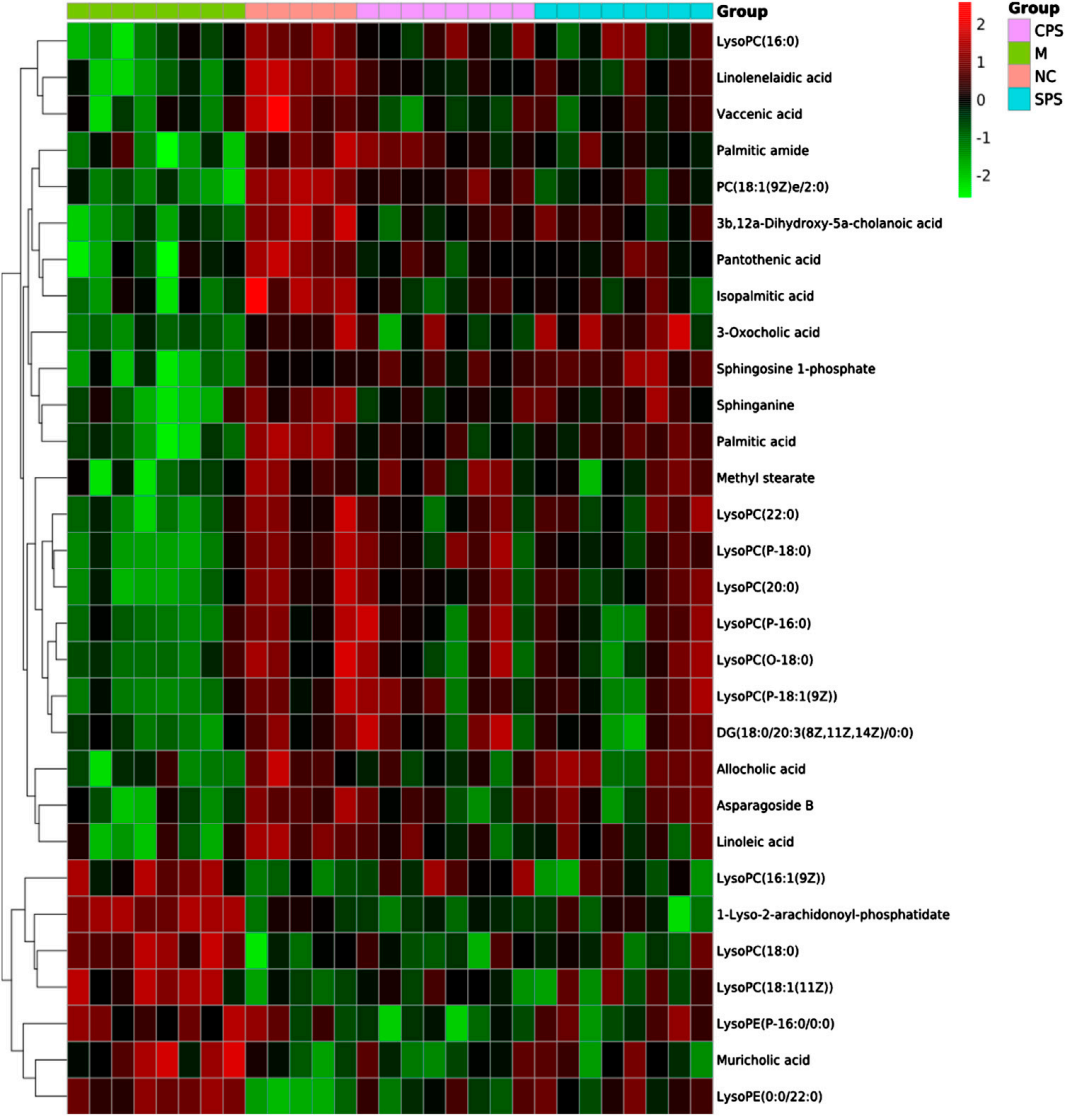
Heat map of the 30 identified differential metabolites. Rows represent these metabolites. Columns represent the corresponding group.

**FIGURE 9:**
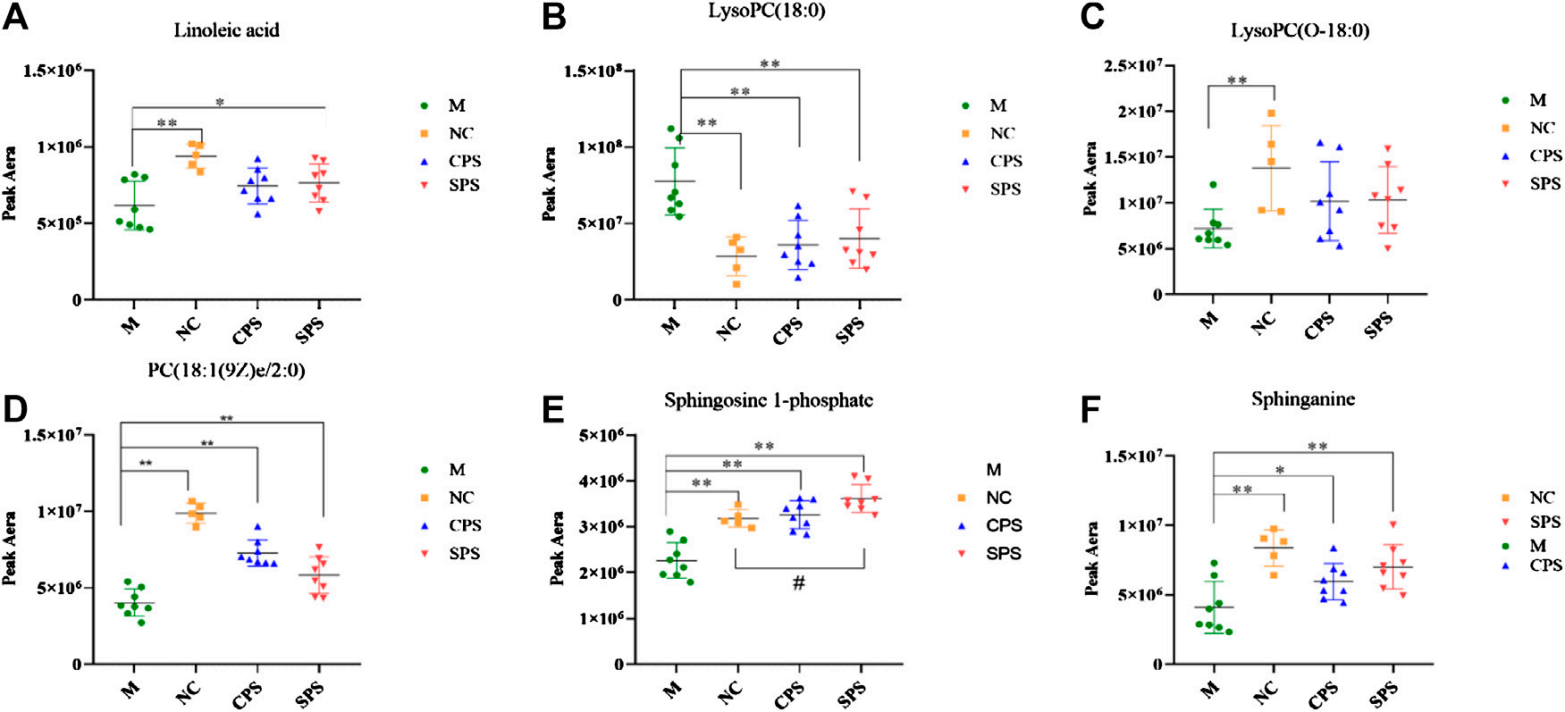
Relative content of common metabolites in the CPS, SPS, and NC groups vs. the M group. ^#^ *p* < 0.05 vs. the NC group; *p < 0.05 vs. the M group; **p < 0.01, vs. the M group. **(A)** Linoleic acid; **(B)** lysoPC(18:0); **(C)** lysoPC(O-18:0); **(D)** PC(18:1(9Z)e/2:0); **(E)** sphingosine-1-phosphate; and **(F)** sphinganine. Six common but major feature metabolites of the CPS and SPS groups were significantly reversed compared to the M group with using a multiple *t*-test and the results were showed in the plots.

A total of 30 putative biomarkers indicating CPS and SPS administration were detected based on S-plot results using accurate *m*/*z* data acquired in positive and negative modes that were matched to the HMDB database. Both the CPS and SPS groups showed similar characteristic metabolites in rats after treatment that correlated with the saline-loaded model (Table 3). This result suggested that the mechanisms of CPS and SPS yielding diuretic effects may be related to these metabolites. In addition, a multiple *t*-test was conducted for the common metabolites. A heat map (Figure 8) depicted different distribution patterns for the 30 metabolites. In addition to the common feature metabolites, such as linoleic acid, palmitoleic acid and lysophosphatidylcholine (LysoPC), 1-lyso-2-arachidonoyl-phosphatidate and linolenelaidic acid were metabolites of the PS group that were also significantly abundant. Notably, sphinganine and sphingosine-1-phosphate were metabolites of SPS that significantly increased compared to the M group (Figure 9). These results demonstrated that CPS and SPS may regulate different metabolic networks to produce diuretic effects. A heat map (Figure 9) depicted different distribution patterns for the 30 metabolites.

#### Metabolic Pathway Analysis

**FIGURE 10:**
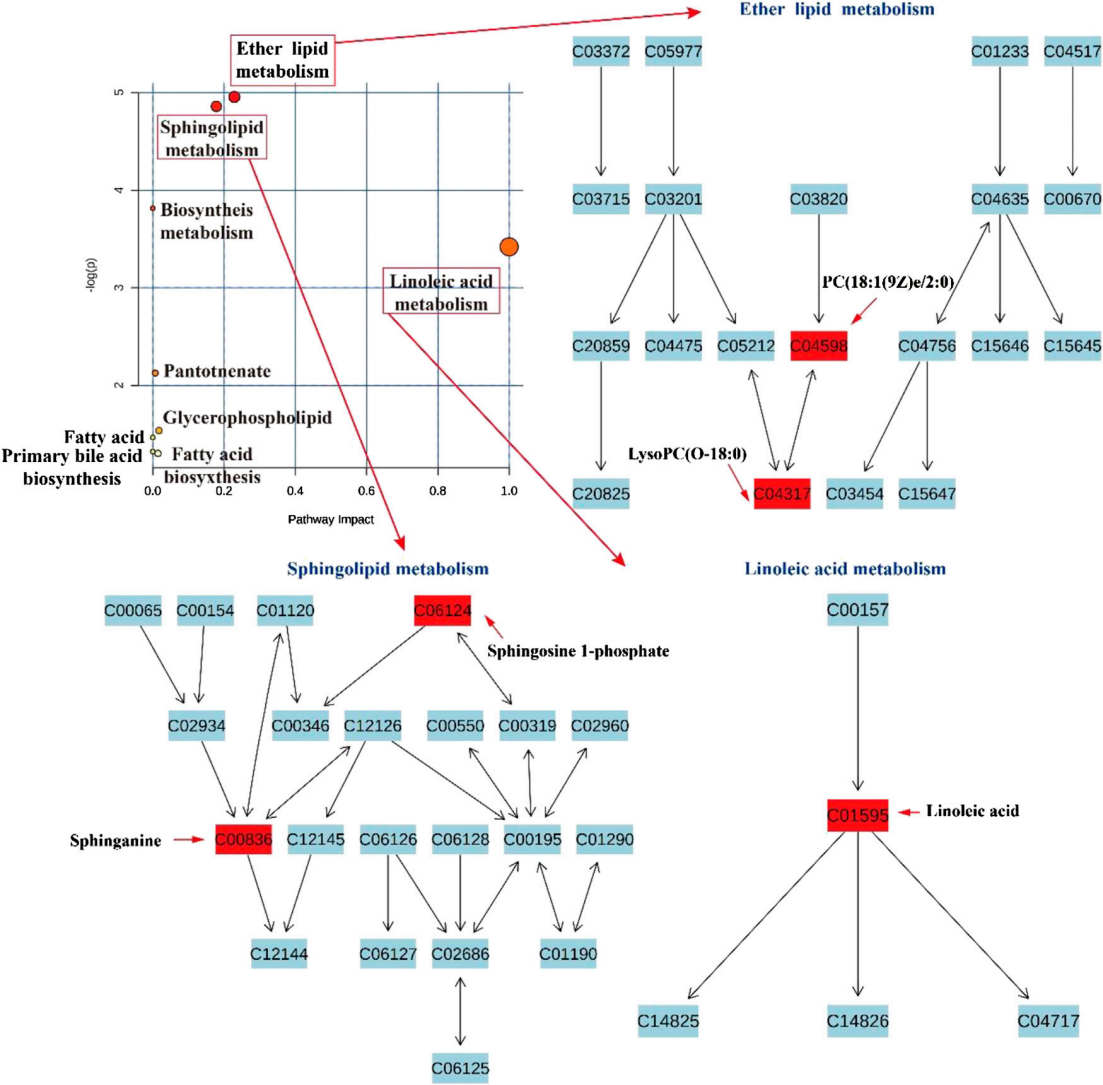
Pathway analysis in saline-loaded rats treated with CPS and SPS.

To investigate the potential mechanism of action of CPS and SPS in saline-loaded rats, at least 30 differentially expressed metabolites were submitted to MetaboAnalyst 4.0 for pathway enrichment. The results indicated that these metabolites are primarily related to the following nine metabolic pathways (impact value >0.1, Figure 10): linoleic acid metabolism (impact value = 1.0), sphingolipid metabolism (impact value = 0.179), and ether lipid metabolism (impact value = 0.229). In addition, CPS acted in saline-loaded rats by regulating the ether lipid metabolism pathway (LysoPC(P-18:0)). SPS had a greater effect on the linoleic acid metabolism pathway (linoleic acid) and sphingolipid metabolism pathways (S1P) than on the ether lipid metabolism pathway of the saline-induced model rats.

**FIGURE 11:**
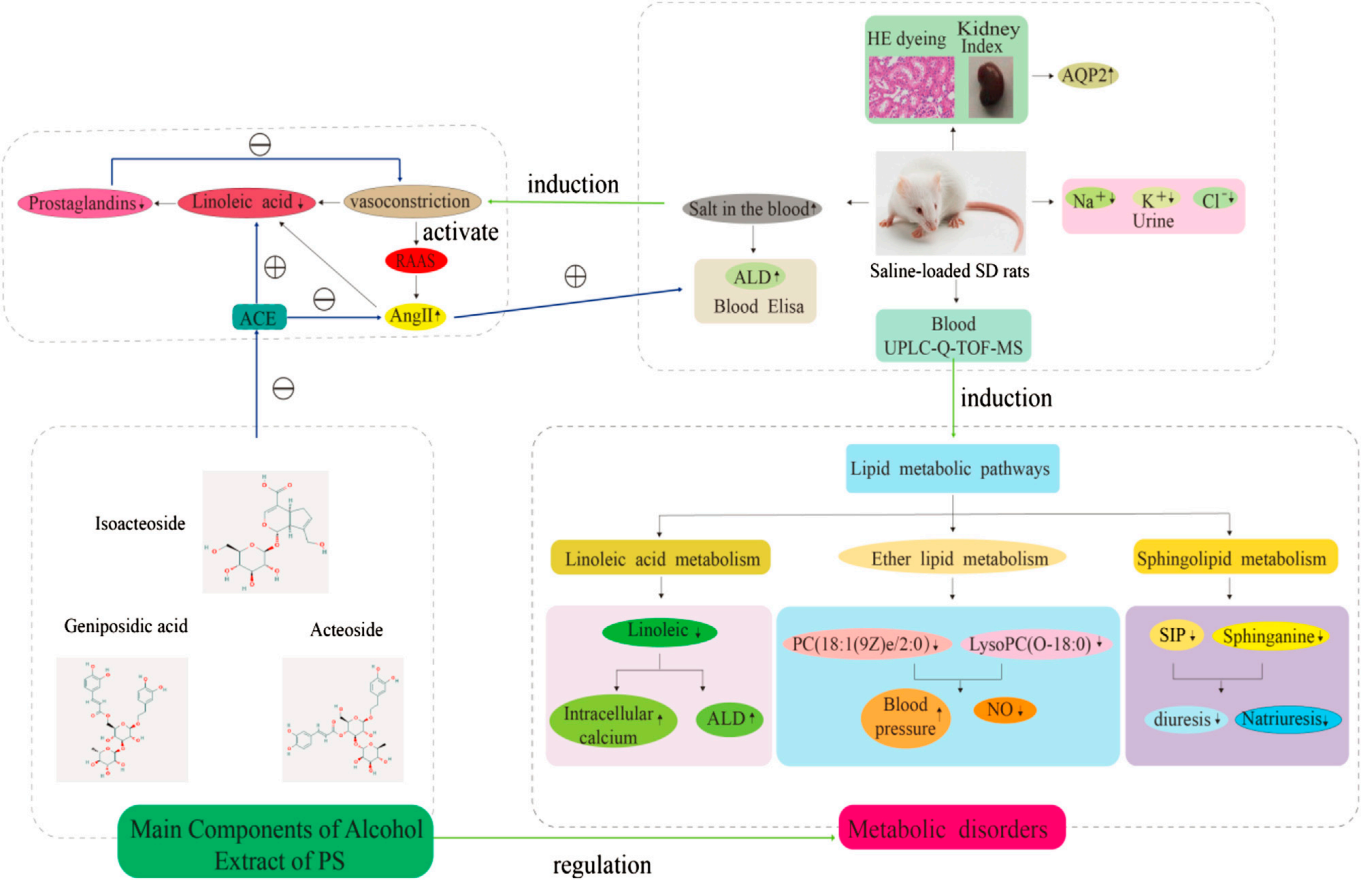
Effects of PS on biochemical indicators, metabolic disturbance in saline-loaded rats. Note: ⊕ indication promotion effect, ⊖ indication inhibition effect.

## Discussion

The number of research studies on natural products to identify new diuretic drugs has significantly increased in the past decade. This is partly due to the gathered information on the role of plant secondary metabolites in renal blood flow (RBF) as well as in enhanced glomerular filtration rate (GFR). Furthermore, medicinal plants with similar diuretic effects have been considered to be potential clinically effective drugs that are less likely to induce side effects (Wright et al., 2007). Thus, this study described the diuretic effect of PS for a model of saline-loaded rats and explored its possible diuretic mechanism and the rationality of salt processing based on a UPLC-Q-TOF/MS metabolomics method. We evaluated the diuretic effect of CPS and SPS on normal SD rats by oral administration, and their pharmacological responses were assessed in relation to furosemide, which is a common diuretic in the clinic, and their impact on electrolyte balance was evaluated. Previous diuretic pharmacology investigations of herbal medicines on saline-loaded normal rats have been performed to assess the diuretic effects of herbal medicines. Furthermore, two parameters, namely, enhanced urine volume (water excretion) and the net decrease in solutes (i.e., electrolytes) in urine, were also evaluated. The current study compared the diuretic impact of CPS and SPS, which were observed to be different. Previous studies have also shown that phenylethanoid glycosides may have a blood pressure-lowering effect (Tong et al., 2019). Other studies have indicated that phenylethanoid glycosides have diuretic effects (Qi et al., 2012). Diuretics are considered the first line of treatment for hypertension, as these increase urine flow and renal sodium excretion, and impart important vasodilator effects (McManus et al., 2012). Here, electrolyte excretion (Na^+^, K^+^, and Cl^−^) markedly increased using 10 g·kg^−1^ doses of CPS and SPS. In this study, the content of the three main components in PS was determined. Compared to CPS, these three indicators of SPS after saline treatment all showed a certain increase. Especially isoacteoside. The UPLC data suggested that phenylethanoid glycosides may be the active chemical component of PS diuresis. Recent works have suggested that taking PS extracts (PSE) daily can regulate the severity of hypertension and thus may be potentially utilized as an antihypertensive herb (Fu et al., 2019). Another study revealed that PSE improves hypertension in spontaneously hypertensive rats by disrupting the activity of angiotensin-converting enzymes (ACEs) (Tong et al., 2019). In addition, we found that there is a relationship between the inhibition of ALD and AQP2 on the diuretic mechanism of PS. ALD is a mineralocorticoid hormone that regulates electrolyte and water homeostasis in the distal tubule. Angiotensin II (AngII) signaling regulates ALD secretion in adrenal glomerulosa cells. Therefore, the renin-angiotensin-aldosterone system (RAAS) plays a central role in establishing electrolyte homeostasis by controlling fluid transport by nephrons (Brown, 2013; MacKenzie et al., 2019). The key process of body water homeostasis is water reabsorption in renal collecting ducts, which is regulated by AQP2 (Noda, 2014). Our results revealed that the levels of the CPS and SPS groups significantly decreased compared to the control group, which may be related to the downregulation of AQP2 expression by PS under (Figure 11).

In this study, UPLC-Q/TOF-MS-based metabolomics was performed to identify distinct metabolites that were related to the occurrence of saline-loaded as well as related metabolic pathways in saline-loaded rats to determine the diuretic effect and underlying mechanism of CPS and SPS. A total of 30 differential metabolites that are involved nine metabolic pathways were detected in rat serum samples using metabolomics. Three metabolic pathways were significantly related to the mechanism of diuresis after PS intervention. According to existing literature reports, linoleic acid metabolism could affect saline-loaded rats in the following two ways. First, lower doses of linoleic acid (C01595) promote ALD secretion (Goodfriend et al., 2002; Goodfriend et al., 2004); ALD is the most potent mineralocorticoid that secreted by the adrenal cortex. When ALD is administered to an animal or human on a high sodium chloride diet, sodium retention and potassium excretion can occur in the kidneys, extracellular fluid volume increases, and arterial blood pressure is elevated. Because of these properties, excessive ALD production has been shown to cause certain types of human hypertension (Burstyn and Horrobin, 1970). Namely, linoleic acid directly caused an increase in ALD content. Second, Ang II induces mitochondrial dysfunction and decreased levels of linoleic acids (Mervaala et al., 2010). This means that saline loading may activate the RAAS, and fatty substances such as linoleic acid can provide energy to the mitochondria to synthesize ACEs, thereby increasing the ALD content. Phenylethanoid glycosides in PS effectively inhibit ACE activation32, which may also be the reason why the linoleic acid in the treatment groups gradually returned to normal levels. CPS and SPS can improve the metabolism of linoleic acid, which was verified with the experimental results of the serum ALD content; that is, the diuretic effect of CPS and SPS (Figure 11).

Sphingomyelin and its metabolites influence vascular tone (Li et al., 2002; Ohmori et al., 2003) and are involved in the mechanism regulating blood pressure (Fenger et al., 2011; Spijkers et al., 2011; Fenger et al., 2015). S1P (C06124) and sphinganine (C00836) are also major biological mediators in various tissues and have been associated with the control of cell growth, differentiation, as well as programmed cell death (Spiegel and Milstien, 2003). In addition, S1P has been reported to enhance diuresis and natriuresis (Bischoff et al., 2001; Czyborra et al., 2006). This enhancement is coupled to alterations in GFR or kaliuresis, implying that S1P imparts diuretic effects in the distal region of the nephron (Bischoff et al., 2010). Given the results of Elisa and metabolites, CPS and SPS target the kidney and possibly act through S1P receptors to regulate sodium excretion by influencing transport mechanisms of the renal medulla, apparently by modulating epithelial sodium channel activity. Additionally, SPS may be more effective at targeting the kidneys than CPS since it underwent salt processing. The internal reasons may be that SPS contains more active ingredients than CPS. Generally speaking, the chemical compositions of drugs are the material basis of pharmacological and pharmacological effects. This increased effectiveness may be another reason why PS should be (Figure 11).

Lower ether lipid synthesis has been associated with various neurological and metabolic abnormalities, whereas increased levels have been linked to cancer, suggesting that proper regulation of ether lipid production is essential to normal physiology (Dean et al., 2018). A previous investigation demonstrated that lower serum ether lipid levels have been associated to hypertension (Graessler et al., 2009), and PC [18:1(9Z) e/2:0](C04598) is an intermediate link between ether lipid metabolism and platelet-activating factor function (Xu et al., 2020). This may imply that the diuretic mechanism of CPS and SPS may be related to lowering hypertension. Linoleic acid metabolism, ether lipid metabolism and sphingolipid metabolism constitute the lipid metabolism pathway, which is concordant to the above results of (Figure 11).

Additionally, although glycerophospholipid metabolism did not seem to be significant, multiple homologs of LysoPC (Kim et al., 2014) were identified in serum samples in the present study. LysoPC is one of the major components of oxidized low-density lipoprotein (ox-LDL) and mildly modified low-density lipoprotein (mm-LDL) production and are synthesized from phospholipids via the lecithin cholesterol acyltransferase (Aoki et al., 2002). LysoPC is one of the major lipid components of the vascular endothelium that is damaged, and it acts by regulating the synthesis and release of nitric oxide (NO) (Quinn et al., 1988; Rikitake et al., 2000). Recent studies have shown that normally intrarenal NO decreases the renal vascular resistance (RVR) and is accountable for up to 30% of the normal RBF, thereby playing a central role in the regulation of natriuresis by disrupting sodium reabsorption in the nephron or by changing the hemodynamics (Majid and Navar, 2001). Thus, NO released by vascular endothelial cells regulates both blood pressure and urination. Other studies have shown that LPC can also induce COX-2 mRNA and protein expression and can thus be utilized in the synthesis of prostacyclin, which is a molecule that possesses vasorelaxant and vasoprotective activity (Zembowicz et al., 1995). Interestingly, the acyl chain length and degree of LPC saturation influences COX-2 and prostacyclin expression. For instance, 16:0 and 20:4 LPC can increase COX-2 expression, whereas 18:1 LPC induces a weak increase in the expression of COX-2 mRNA (Brkić et al., 2012). These were consistent with the results obtained.

In summary, we expectedly found a series of metabolic changes in saline-loaded rats after administration of CPS and SPS, which may be part of the underlying mechanisms of the diuretic effect of PS alcohol extracts and may promote PS processing. Moreover, CPS and SPS have imparted good regulatory effects on endogenous metabolites that are mainly involved in the above three pathways. Although we observed the diuretic effect of PS alcohol extracts and elucidated the relevant mechanism and processing mechanism by metabolomics, there are several limitations to the present study: 1) we could not comprehensively detect all metabolites in a specific metabolic pathway by UPLC-Q/TOF-MS, so multi analytical techniques need to be implemented; 2) on the other hand, given our limited understanding of some lipid metabolites, the significance of these lipids *in vivo* remains unknown, and we selected only representative substances in related metabolic pathways for interpretation; and 3) the active ingredients that had diuretic effects were confirmed only by the increase in the content of the main components after processing and by the related literature. Future studies should explore the mechanistic basis of these metabolites in diuretic TCM materials.

## Conclusion

In conclusion, both CPS and SPS have diuretic effects that are mainly produced by regulating related products in the three major metabolic pathways and the RAAS. The diuretic effect of SPS, which is saline-processed, was better than that of CPS. In addition, serum biochemical, histopathological, and metabolomic strategies were combined to explore the potential *in vivo* targets of CPS and SPS, present references for in-depth mechanistic explorations, as well as to improve the applications of PS to the clinic, and provide a reference for research studies on other TCMs.

## Data Availability Statement

The raw data supporting the conclusions of this article will be made available by the authors, without undue reservation, to any qualified researcher.

## Ethics Statement

The animal study was reviewed and approved by Animal Care and Research Committee of Jiangxi University of Traditional Chinese Medicine (Nangchang, China) under license NO. JZLLSC2020_0108.

## Author Contributions

CL and DL designed the experiments. CL and RW analyzed the data and wrote the manuscript. RW completed the metabolomic detection. LY and JW completed the experiments of saline-loaded model. YG and SL helped with data analysis. CL and QL revised the manuscript HY and QG refined the manuscript for publication. All authors read and approved the final manuscript.

## Funding

This work was supported by the National Standardization Project of Traditional Chinese Medicine (ZYBZH-Y-JX-27).

## Conflict of Interest

The authors declare that the research was conducted in the absence of any commercial or financial relationships that could be construed as a potential conflict of interest.
