# Supplementary material for: Diuretic Effect and Metabolomics Analysis of Crude and Salt-Processed Plantaginis Semen
Source: Front Pharmacol. 2020 Nov 24;11:563157. doi: 10.3389/fphar.2020.563157 (PMC7774519; doi:10.3389/fphar.2020.563157)
Supplement: Supplementary file 1 [file Image1.pdf]

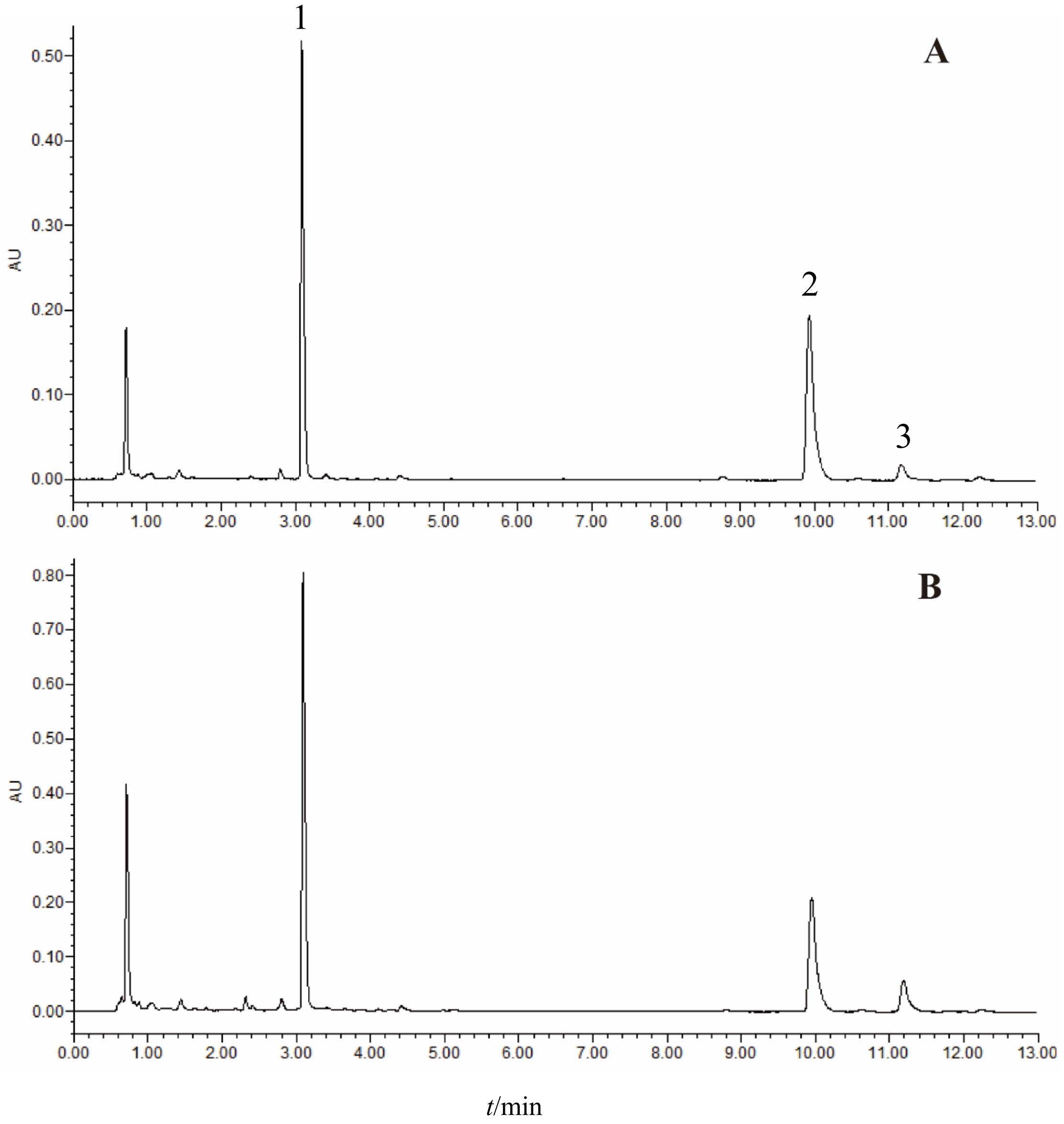

Figure S1| UPLC chromatograms of PS decoctions at 254 nm wavelength: A-CPS, B-SPS

1—geniposidic acid, 2—verbascoside, 3—isoverbascoside
